# Using Iron Tailings for Phosphate Removal in Cemented Phosphogypsum (PG) Backfill

**DOI:** 10.3390/ma15238497

**Published:** 2022-11-29

**Authors:** Ying Shi, Xiaolin Wang, Zixuan Qing, Yanmei Song, Jie Min, Yanan Zhou, Jing Du, Shaofeng Wang

**Affiliations:** 1 School of Resources and Safety Engineering, Central South University, Changsha 410083, China; 2 School of Materials Engineering, Changshu Institute of Technology, Changshu 215500, China; 3 Suzhou Sinoma Design and Research Institute of Non-Metallic Minerals Industry Co., Ltd., Suzhou 215151, China

**Keywords:** phosphogypsum, cemented backfill, iron tailings, phosphate removal by adsorption

## Abstract

Compared with the post-treatment of pollutants, such as the removal of phosphate from wastewater, it is more important to develop effective emission control strategies to reduce phosphate pollution. Phosphogypsum (PG) is a typical solid waste byproduct of phosphate production and contains high amounts of residual phosphate. In order to control the phosphate emissions during the recycling of PG aggregates for cemented backfill, another solid waste product—iron tailings (ITs)—was added during the preparation of backfill slurry. The results showed that the ITs effectively accelerated the phosphate removal in cemented PG backfill, enabling the quick reduction in the phosphate concentration to the discharge standard (<0.5 mg/L) within 15 min. This means that the emissions of phosphate to bleeding water were effectively controlled. The adsorption experiment showed that phosphate was adsorbed by the ITs, and the adsorption data fitted well with the Langmuir adsorption model *(R*^2^ = 0.98) and pseudo-second-order kinetic model (*R*^2^ = 0.99), indicating that the phosphate adsorption of ITs was a monolayer chemical adsorption. Furthermore, an unconfined compressive strength (UCS) test was performed on the backfill with the addition of ITs. Compared to the control group (without ITs), the UCS of backfill with 20% ITs increased from 1.08 MPa to 1.33 MPa, indicating that the addition of solid waste could be beneficial to the strength development of the backfill by mitigating the interference of phosphate with the hydration process. The backfill cured for 28 d was selected for the toxic leaching test, and the phosphate concentration in the leachates was always below 0.02 mg/L, indicating that ITs can effectively immobilize phosphate in backfill for a long time.

## 1. Introduction

Secondary pollution should be fully considered in the recycling of solid waste [1]. Phosphogypsum (PG) is a typical solid waste byproduct in the production of phosphoric acid; it is mainly composed of calcium sulfate dihydrate, and also contains high concentrations of soluble phosphate and other impurities (2–4%) [2,3,4]. Some technologies of PG utilization have been developed. For example, PG is reused as building materials to produce building gypsum powder and the additive to cement [5,6]. PG is also used as fertilizers or soil stabilization amendments in agriculture [7,8]. Furthermore, the PG can be a resource to be considered to recover rare earth elements [9,10,11]. Cemented backfill technology with PG as an aggregate can massively recycle PG, solving the problem of PG surface storage; however, the high concentration of phosphate will be released again into the bleeding water of the backfill slurry and the underground leachates, causing secondary pollution and threatening the ecological environment [12,13,14]. Therefore, the efficient removal of phosphate from cemented PG backfill is an issue that cannot be ignored.

At present, there are several methods for removing phosphate, such as chemical precipitation, biological treatment, adsorption, and membrane separation [15,16,17,18]. Among them, adsorption has attracted extensive attention due to its simple operation, less residue generation, strong adaptability, and the ability to remove phosphate at low concentrations [19,20,21]. Zhang et al. [22] prepared a novel type of Fe–Mg–Zr layered bimetallic hydroxide (LDH) via a simple in situ crosslinking method and applied it to wastewater containing phosphate. The maximum phosphate adsorption capacity of the LDH was 21.61 mg/g, and it could still be used after five desorption cycles. Hu et al. [23] prepared a hierarchical CuAl/biomass carbon fiber LDH for phosphate removal, and the removal rate reached up to 99.6% within 50 min. However, most of the materials used for adsorption are expensive, so the development of low-cost materials that can be used for the removal of phosphate via adsorption has attracted intensive interest [24,25]. Li et al. [26] found that the maximum capacity of the drinking-water treatment residuals (DWTRs) to adsorb phosphate reached 19.6 mg/g, which is mainly due to the great amounts of amorphous iron or aluminum in DWTRs. Letshwenyo et al. [27] studied the ability of copper smelting slag to remove phosphate from wastewater through batch experiments; the theoretical and experimental phosphate adsorption capacity reached 0.16 mg/g and 0.26 mg/g, respectively. Obviously, the use of waste and industrial byproducts for phosphate removal can greatly reduce the cost.

Iron tailings (ITs) are a kind of solid waste with a low content of valuable elements produced during the exploration of iron resources. It can be used for the production of road materials, building brick, trace-element fertilizers and concrete [28,29,30,31]. Furthermore, ITs have become a research hotspot among many scholars due to their superior phosphate adsorption performance and low cost. Zeng et al. [32] studied the adsorption isotherm, adsorption kinetics, desorption, and other relevant properties of ITs with respect to phosphate. It was verified that ITs can act as an adsorbent of phosphate with superior performance (4.6 mg/g), and the adsorption was irreversible. Sima et al. [33] found that ITs can remove phosphate from wastewater at a rate of up to 1.29 mg/g. Furthermore, ITs have great regeneration potential, as their phosphate removal rate is still 56% after three cycles, and they can be used as a good phosphate adsorbent.

However, most of the previous studies used ITs for the removal of phosphate from sewage, and there has been no research on the use of ITs for controlling phosphate pollution in cemented PG backfills. In fact, compared to the treatment of phosphate after the drainage of bleeding water, it is more meaningful to control phosphate emissions from backfill slurry. Therefore, this study aimed to study the feasibility of using ITs for the immobilization of phosphate in cemented PG backfills. With the addition of various amounts of ITs during the preparation of the backfill slurry, the adsorption rate and the adsorption mechanism were studied. In addition, the effects of ITs on the performance of backfills were tested by measuring the pH, initial setting time and final setting time, and unconfined compressive strength (UCS) of the backfill. Finally, toxic leaching tests were performed on the backfill after curing for 28 d to evaluate the long-term effects of ITs on the immobilization of phosphate.

## 2. Materials and Methods

### 2.1. Materials

**Figure 1:**
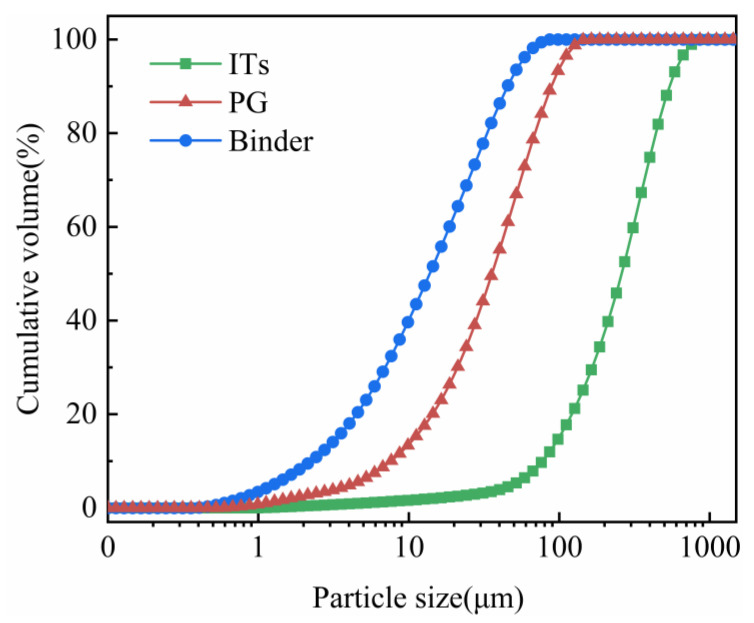
Particle size distribution of PG, binders and ITs.

The ITs used in the present experiments were collected from a mine in Yunnan, China, and washed thrice with deionized water to remove the adhered dust and soluble substances. After washing, they were oven-dried for 24 h at 105 ℃. The PG and binder were collected from a phosphate fertilizer company in Guiyang, China. The PG was a moist gray–black powder with a pH of 1.9. The binder was composed of yellow phosphorous slag, fly ash and cement clinker in 6:1:3 ratio [34]. The particle size distribution of PG, ITs and binder are shown in Figure 1.

**Figure 2:**
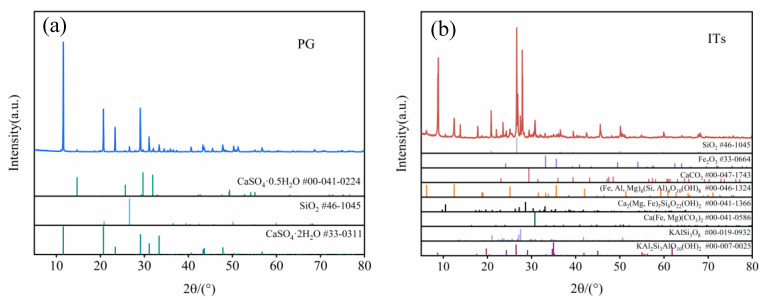
X-ray diffraction patterns of PG (**a**) and ITs (**b**).

The diffraction patterns of the ITs and PG were characterized via X-ray diffraction (XRD) using a D8 X-ray automatic diffractometer (Bruker, Billerica, MA, USA) with a resolution of 0.02°/s in the angular domain 5–80°, and the results are shown in Figure 2. The chemical composition of ITs, PG and binder were measured through an X-ray fluorescence analyzer (Bruker, Billerica, MA, USA), as shown in Table 1.

**Figure 3:**
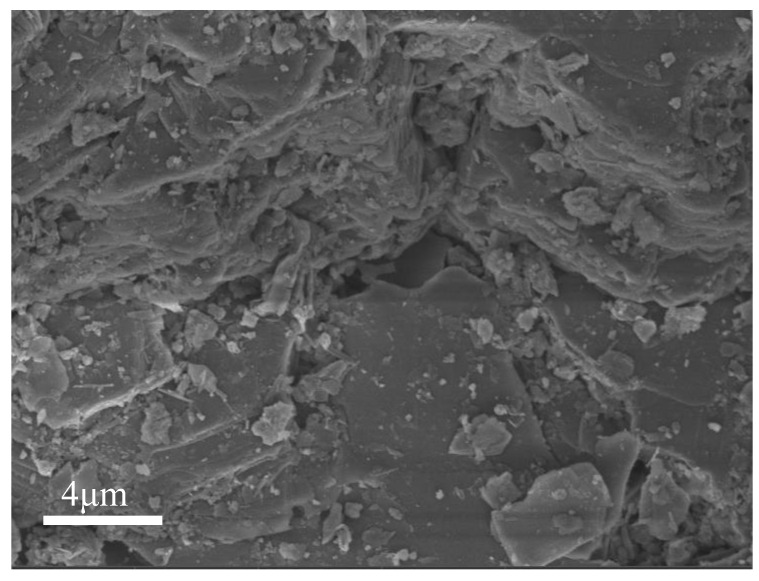
SEM image of ITs.

A scanning electron microscope equipped with an energy-dispersive X-ray spectroscope (SEM-EDS) was used to characterize the ITs and backfill. The microstructure and surface elemental content of the materials were analyzed using a TESCAN MIRA4 scanning electron microscope (TESCAN, Brno, Czechia). Figure 3 shows the SEM image of the surface morphology of the ITs. It can be seen that the surface of the ITs is rough, with irregular distribution of lumps and gaps. This surface morphology means that the ITs have a large specific surface area and high porosity, providing a great amount of active adsorption sites for phosphate removal.

According to previous studies, the reaction of functional groups on the surface of substances—caused by van der Waals forces—is the reason for the adsorption of the molecules [35]. Therefore, the chemical properties of ITs play an important role in their ability to adsorb phosphate. The XRD result show that ITs (Figure 2b) contained (KAl_2_(AlSi_3_O)(OH)_2_), Fe_2_O_3_ and Ca(MgFe)(CO_3_)_2_;. The XRF result shows that ITs (Table 1) contained 11.47% of Fe_2_O_3_, 12.98% of Al_2_O_3_, and 4.68% CaO, respectively. Iron (Fe^3+^), aluminum (Al^3+^), calcium (Ca^2+^), and other metal ions contained in ITs have an affinity for phosphate [36]. Therefore, the ITs have the necessary chemical conditions for the adsorption or complexation of phosphate. 

### 2.2. Preparation of Cemented PG Backfill Slurry 

To investigate the change in phosphate concentration in the cemented backfill, the backfill slurry was prepared with the mixing proportions shown in Table 2. PG aggregate, binder, and water were added to a PP container, where the dry mass ratio of PG to binder was 5:1, and the liquid–solid ratio was 5:1. Three batches of backfill slurry were prepared. After being mixed uniformly, each batch of slurry was immediately divided into two parts: one was combined with various contents of ITs as the experimental group, and the other part was considered as the control group (PBS). Three ITs mixtures (5, 20, and 35 wt.% PG) were tested in this study, denoted as PBSI-5, PBSI-20, and PBSI-35, respectively. Taking the time at which the ITs were added as the zero point, the experimental and control groups were sampled at mixing times of 0, 1, 2, 3, 4, 5, 15, 45, and 105 min. Immediately after sampling, the slurry was filtered through a 0.45 µm microporous membrane to obtain the bleeding water from the backfill slurry for the analysis of pH and phosphate concentration.

### 2.3. Phosphate Adsorption Experiment

#### 2.3.1. Adsorption Isotherms of Phosphate on ITs

In order to explore the phosphate adsorption process of ITs, adsorption isotherm experiments were carried out. First, 3 g of ITs was weighed and added to a 100 mL conical flask, and then 50 mL standard solutions of KH_2_PO_4_ at different concentrations (i.e., 0.5, 2, 5, 10, 15, 25, and 50 mg/L) were added to the flask. At room temperature, the conical flask was placed in an oscillating device and vibrated for 24 h at 150 rpm HCl or NaOH was used to maintain the pH of the solution at 7. Then, the mixture was filtered through a 0.45 µm microporous membrane, and the filtrate was used to measure the phosphate concentration. The data were fitted to the Langmuir and Freundlich isotherm models to analyze the adsorption equilibrium parameters and adsorption behavior.

#### 2.3.2. Adsorption Kinetics of Phosphate on ITs

To study the adsorption kinetics, 3 g of ITs and 150 mL of KH_2_PO_4_ solution with a phosphate concentration of 20 mg/L were added to a 250 mL conical flask. The pH of the mixed solution was maintained at 7 with HCl or NaOH. The conical flask was placed in an oscillating device at room temperature and shaken at 150 rpm. The supernatant of the sample was filtered after 1, 2, 4, 8, 12, 24, and 30 h. Then, the filtrate’s phosphate concentration was measured. The data were fitted to the pseudo-second-order model to determine the phosphate removal kinetics model.

### 2.4. Preparation of Cemented PG Backfill

In order to explore the influence of ITs on the strength development of the cemented PG backfill, backfill with various amounts of ITs was prepared with the mixing proportions shown in Table 3. The mass ratio of aggregate (PG + ITs) to binder was 4:1, and the solid mass concentration was 60%. Four batches of backfill were prepared with different proportions of ITs to PG, i.e., 0, 5, 20, and 35% (PB, PBI-5, PBI-20, and PBI-35, respectively). PG, binder, ITs, and water were first added to a PP container. After mixing for 30 min, the slurry was poured into a plastic mold with a length, width, and height of 40 mm. After the final setting of the backfill, the samples were demolded and placed in a curing box at a constant temperature of 20 ± 2 ℃ and a humidity of 90 ± 5%.

### 2.5. Unconfined Compressive Strength Tests

In order to evaluate the impact of adding ITs on the mechanical properties of the backfill, UCS tests were performed for backfill samples cured for 7 d, 14 d, and 28 d. According to the JGJ/T 70-2009 standard [37], the test was carried out using a WHY-200/10 servo pressure testing machine (Hualong, Shanghai, China) with a loading rate of 0.1 mm/min. Each backfill sample had three replicates, and the average value was used for the calculation of UCS at different curing ages.

### 2.6. Toxicity Leaching Test

Cemented PG backfill is transported to underground stopes where it is in long-term contact with groundwater [13]. Therefore, it is necessary to determine whether the phosphate can be fixed in backfill for an extended period. Toxicity leaching tests were carried out on the backfill according to the Chinese standard HJ557-2010 [38]. The backfill cured for 28 d was ground and put in an oven for drying at 55 ℃. Then, the dried backfill was mixed with deionized water at a liquid–solid ratio of 10:1 in a PP bottle and shaken on a horizontal vibrator at 110 rpm for 8 h. Finally, it was left to stand for 16 h, and the supernatant was filtered to measure the phosphate concentration. Each test was performed in triplicate to ensure the accuracy of the measurement.

### 2.7. Chemical Measurements 

The phosphate concentration measured in this study was the concentration of soluble orthophosphate (PO_4_^3−^-P), which was determined via the ammonium molybdate tetrahydrate spectrophotometry method. First, 100 µL of 10% ascorbic acid solution was added to 5 mL of the sample. After the mixture was shaken well, 200 μL of H_8_MoN_2_O_4_ was added in solution. After standing for 15 min, the solution was put into a Shimadzu UV1800 spectrophotometer (Shimadzu, Kyoto, Japan) and measured at the wavelength of 700 nm. The pH of the backfill slurry was measured using a Mettler Toledo FE28 pH meter (Shanghai, China).

## 3. Results and Discussion

### 3.1. Phosphate Adsorption of Its in Cemented PG Backfill

#### 3.1.1. Effect of the Addition of ITs on the Removal of Phosphate from the Backfill Slurry

**Figure 4:**
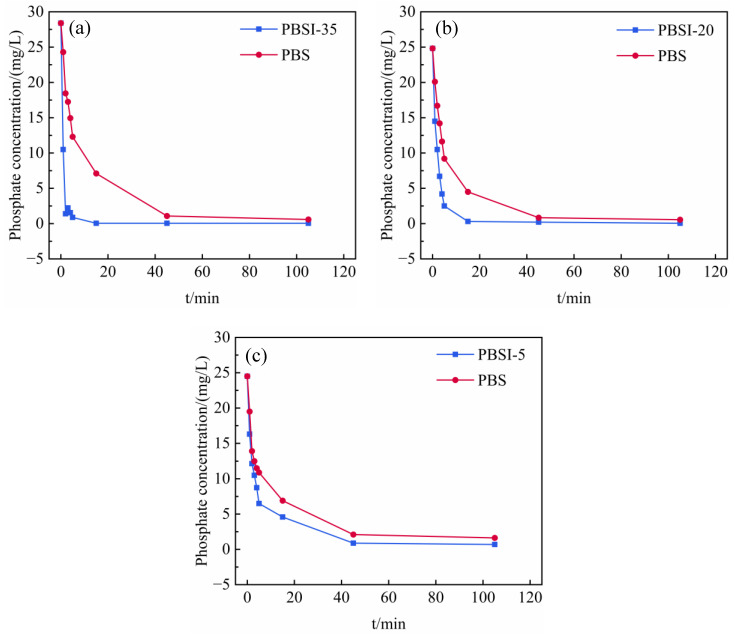
Changes in the phosphate concentration in backfill slurry with different amounts of ITs: (**a**) 35% ITs addition; (**b**) 20% ITs addition; and (**c**) 5% ITs addition.

When the ITs were added to the backfill slurry at proportions of 5%, 20%, and 35%, the changes in the phosphate concentration were monitored, as shown in Figure 4. The initial concentration of phosphate in the backfill slurry was 25–28 mg/L. Without the addition of IT (PBS), the phosphate concentration in the slurry decreased gradually. This was because the hydration reaction of the binder produced a large amount of Ca^2+^, which combined with the phosphate to form precipitates such as calcium phosphate salt [39,40]. In comparison, with the help of ITs, the rate of decrease in the phosphate concentration was more remarkable. For example, as shown in Figure 4a, the phosphate concentration in the control group changed from 24.5 mg/L to 19.5 mg/L in the first minute, decreasing by 20.4%. However, when the amount of ITs added was 5%, the concentration of phosphate changed from 24.5 mg/L to 16.3 mg/L in the first minute, decreasing by 33.5%, indicating that phosphate was adsorbed more quickly due to the addition of ITs. The phosphate removal rates in the other groups (PBSI-20 and PBSI-30) were also faster than the control group. This indicates that the ITs were involved in phosphate adsorption and could remove phosphate from the slurry in a short time.

The amount of ITs added affected the solidification of phosphate in the backfill. During the whole mixing period of 2 h, the phosphate concentrations of the PBS and of the lowest proportion of ITs (i.e., PBSI-5) were 1.63 mg/L and 0.70 mg/L, respectively, which meant that the phosphate concentration in the bleeding water exceeded the discharge standard (<0.50 mg/L) [41]. However, the phosphate concentration of PBSI-20 descended to 0.31 mg/L at 15 min, which was lower than the discharge standard. When the proportion of ITs was increased to 35%, the phosphate removal rate was even higher, and the phosphate concentration was 0.05 mg/L, while the corresponding control group phosphate concentration was 7.10 mg/L after 15 min of mixing. This shows that in the cemented PG backfill, increasing the proportion of ITs can effectively increase the phosphate removal rate and control the phosphate concentration in the bleeding water.

Based on the above results, during the preparation of the backfill slurry, adding sufficient ITs could quickly and effectively solidify the phosphate, resulting in the phosphate concentration in the bleeding water being below the discharge limit. This means that the post-treatment of wastewater could be avoided, indicating that adsorption by ITs in backfill preparation is an effective emission control strategy for phosphate pollution. This provides a new direction for the removal of phosphate from cemented PG backfill. Furthermore, this method achieves the disposal of waste using other waste, representing a reproducible approach for the large-scale recycling of solid waste.

#### 3.1.2. Effect of ITs on the pH of Backfill Slurry

**Figure 5:**
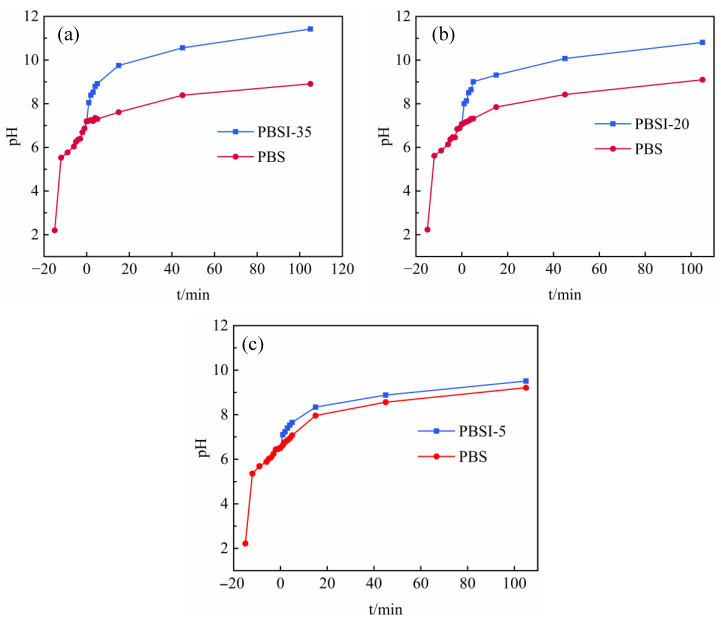
Changes in pH in backfill slurry with different amounts of ITs: (**a**) 35% ITs addition; (**b**) 20% ITs addition; and (**c**) 5% ITs addition.

Figure 5 shows the changes in the pH of the backfill slurry with mixing time. When the low-pH PG aggregate was mixed with the binder, the pH increased gradually from 2.2 to 7–7.3 within 20 min, which was related to the increase in the amount of OH^-^ due to the hydration reactions of the binder [42]. When the ITs were added to the backfill slurry, the pH increased to even higher levels. After adding 5% ITs, the pH of the slurry increased from 6.35 to 6.67, representing an increase of 5.03%, while the pH of the control group increased from 6.35 to 6.55, representing an increase of 3.14%. The pH of PBSI-35 increased from 7.19 to 8.05, representing an increase of 11.96%, while the corresponding control group only increased from 7.19 to 7.22, representing an increase of only 0.42%. One reason for this is that after adding ITs, the calcium carbonate and metal oxides in the ITs (Figure 2b) can consume some of the hydrogen ions in PG and, thus, increase the pH [43]. Another reason is that ITs can adsorb phosphate. When the pH is within the range of 7–10, the phosphate in the solution mainly exists as H_2_PO_4_^−^ and HPO_4_^2−^ [44]. When the phosphate was adsorbed by the ITs, according to the principle of ionization balance, the hydrogen ions in the solution decreased and the pH increased in the backfill slurry [45]. As shown in Figure 4 and Figure 5, the more ITs added, the faster the decline in the pollutant concentrations and the greater the pH of the slurry. Therefore, the higher the amount of ITs added, the higher the pH of the backfill slurry.

#### 3.1.3. Adsorption Mechanism of Phosphate by ITs in Backfill

The adsorption kinetic model is usually used to estimate the adsorption rate, which is an important feature to judge the adsorption performance of an adsorbent. Pseudo-first-order and pseudo-second-order kinetic models have been widely used to describe adsorption systems [46,47]. According to the research of Sima et al. [33], ITs are more consistent with the pseudo-second-order kinetic equation. Therefore, the following equation was used to fit the experimental data [48]:

(1) $$\frac{t}{Q_{t}}=\frac{1}{K_{2}Q_{e}{}^{2}}+\frac{t}{Q_{e}}$$

where *Q_t_* is the amount of phosphate adsorbed in time *t* (mg/g), *Q_e_* is the equilibrium adsorption amount (mg/g), and *K*_2_ is the adsorption equilibrium rate constant of the pseudo-second-order equation (g(mg/h)).

**Figure 6:**
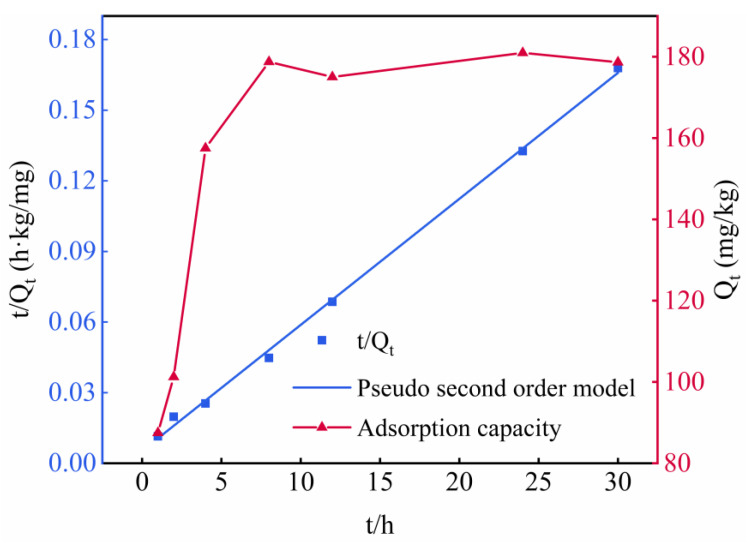
Pseudo-second-order kinetic model and adsorption rate curve of ITs.

The curve of the adsorption rate of phosphate by ITs was evaluated, and the results are shown in Figure 6. It was observed that the removal of phosphate increased very quickly at first, and then gradually approached constant values. To be specific, the rate of phosphate adsorption was fast in the first 5 h of the adsorption reaction; then, the adsorption rate decreased and reached equilibrium slowly within 10 h. It is possible that with the increase in time, the pores and cation sites of ITs are gradually occupied by phosphate, resulting in no sites being available for phosphate [49]. 

The fitting of the pseudo-second-order model of ITs is represented in Figure 6. The correlation coefficient (*R*^2^) of the pseudo-second-order kinetic equation is 0.99, indicating that the phosphate adsorption process of ITs is consistent with the pseudo-second-order kinetic model. This illustrates that the adsorption process is chemical adsorption and that there is a chemical bond between the ITs’ surface and the phosphate [50], and the adsorption is relatively stable, with the equilibrium amount of adsorbed phosphate being 185.19 mg/kg. When the amount of ITs added is 70 g and 40 g, the theoretical amount of adsorbed phosphate is 12.9 mg and 7.4 mg, respectively. According to Figure 1, the actual decrease in the amount of phosphate in the backfill slurry with 70 g and 40 g of ITs was about 15.6 mg and 6.7 mg, respectively, which are close to the theoretical values.

**Figure 7:**
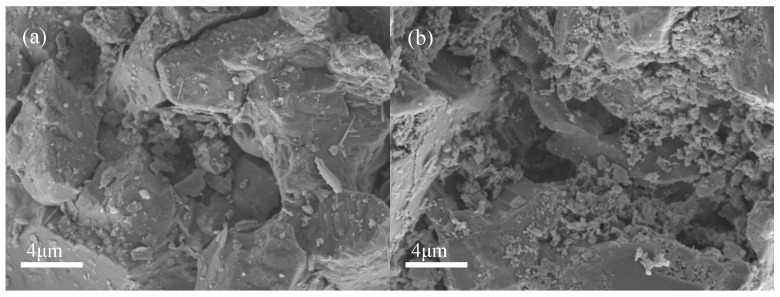
SEM images of ITs before (**a**) and after (**b**) phosphate adsorption.

Figure 7 shows a comparison of the micromorphology of ITs before and after adsorption in the kinetic experiment. Wang et al. [45] showed that phosphate was adsorbed by iron oxide in ITs through surface complexation and produced iron-based phosphate products. In this study, before adsorption, the surface of the ITs was rough, with a porous structure and many gaps. After adsorption, there were many fine and irregular particles appearing on the surface and pores of the ITs. This might be due to phosphate ions precipitated with ferric iron of ITs forming low-solubility iron–phosphate precipitates [51,52]. Additionally, the protonation of the adsorbent surface in the aqueous solution could make it adsorb phosphorus anions by electrostatic adherence, which was further immobilized by ligand exchange [45]. These indicated that the phosphate in PG was effectively adsorbed by the ITs.

The adsorption isotherm describes the adsorption processes at the solid–liquid interfaces and can be studied to calculate the adsorption capacity and explain the adsorption mechanism [53,54]. In this study, the adsorption data were fitted according to the Langmuir and Freundlich isotherm models. The linear form of the Langmuir isotherm model can be expressed as follows [55]:

(2) $$\frac{C_{e}}{Q_{e}}=\frac{1}{K_{L}q_{m}}+\frac{C_{e}}{q_{m}}$$

where *Q_e_* is the adsorption capacity of the adsorbent at adsorption equilibrium (mg/kg), *q_m_* is the adsorption capacity when the adsorbent reaches saturation (mg/kg), *K_L_* is the Langmuir equilibrium constant (L/mg), and *C_e_* is the concentration of the adsorbed ion when the adsorption in the solution reaches equilibrium (mg/L). In addition, from the Langmuir equation, a separation factor (*R_L_*) can reflect the feasibility of the adsorption process. This can be expressed as follows:

(3) $$R_{L}=\frac{1}{{1+K}_{L}C_{0}}$$

where *C*_0_ is the initial P concentration (mg/L). The correlation coefficient *R_L_* of the Langmuir equation is equal to 1, indicating that the adsorption is linear; when the *R_L_* value is less than 1, it means that it is favorable for adsorption, while *R_L_* > 1 indicates that it is not conducive to adsorption [56].

The linear form of the Freundlich isotherm model can be expressed as follows [57]:

(4) $${\mathrm{logq}}_{e}{=\mathrm{logK}}_{F}+\frac{1}{n}{\mathrm{logC}}_{e}$$

where *K_F_* is the Freundlich constant (mg/kg) and 1/*n* is the adsorption strength parameter.

**Figure 8:**
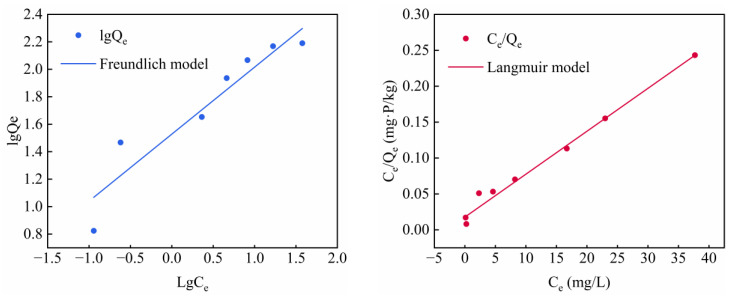
Langmuir and Freundlich models for the adsorption of phosphate onto ITs.

The fitting results of the experimental data based on the two adsorption models are shown in Figure 8, and the relevant parameters of the Langmuir and Freundlich isotherms are shown in Table 4. The Langmuir and Freundlich correlation coefficients are both larger than 0.89; this shows that both models can explain the adsorption process, indicating that there are both homogeneous and heterogeneous adsorption sites on the ITs. However, the Langmuir correlation coefficient (*R*^2^ = 0.985) is higher than the Freundlich correlation coefficient (*R*^2^ = 0.898), indicating that the Langmuir isotherm model is more suitable for the adsorption process of ITs. The adsorption of phosphate on Its mainly takes place in a single, uniform layer [58]. In addition, in this experiment, *R_L_* was between 0 and 1, indicating that it is conducive to adsorption. According to the curve-fitting results, the equilibrium adsorption capacity of the ITs is 163.93 mg/kg, which is also close to the results of the adsorption kinetics.

The adsorption isotherm and kinetics studies show that the adsorption process of phosphate on ITs is mainly monolayer chemical adsorption. Previous studies have shown that the adsorption process of phosphate on ITs is mainly attributable to the Fe_2_O_3_ in ITs and includes ligand exchange, precipitation, and electrostatic adherence [45]. In addition to Fe_2_O_3_, Al^3+^, Ca^2+^, and other components in ITs may also promote the removal of phosphate through adsorption or precipitation to some extent [59]. These chemical bonds are formed between the medium surface and the adsorbate, so the adsorption is relatively stable and is not easy to reverse [60]. Combined with the removal of phosphate via precipitation by hydration reactions of the binder during the mixing of the backfill slurry, the cemented PG backfill could effectively and quickly remove phosphate, enabling the phosphate concentration in bleeding water to meet the discharge standard (GB8978-1996).

**Figure 9:**
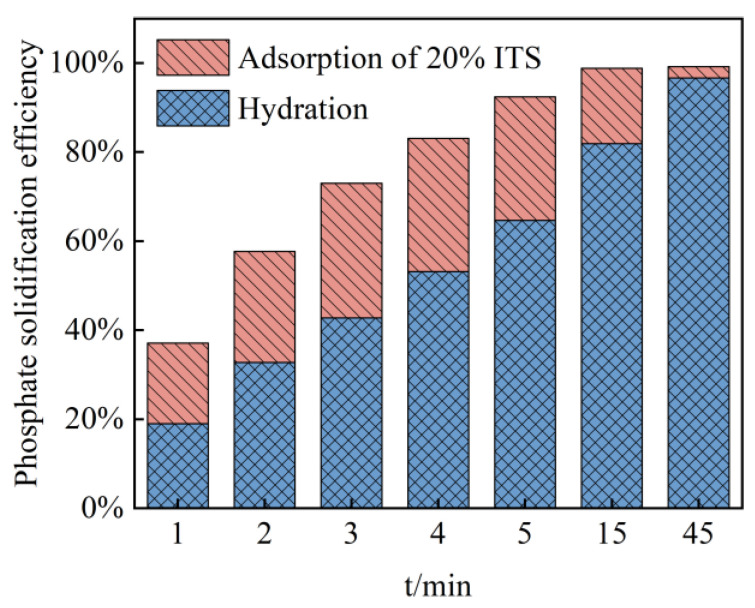
Removal rate of phosphate from slurry by ITs and hydration reaction.

The changes in phosphate removal efficiency and the contribution of ITs were calculated, as shown in Figure 9. Under the joint action of the binder and ITs, the phosphate removal efficiency increased to 99% within 15 min of mixing (defining the final phosphate removal efficiency as 100% after two hours of mixing). At the first minute, the total removal efficiency was 41%, half of which was attributed to the adsorption by ITs. In the next several minutes, the phosphate removal efficiency attributed to adsorption by ITs remained at 20–35%, while the hydration contributed more and more until the removal efficiency reached 100% at 15 min. Then, with the saturation of ITs’ adsorption, hydration gradually began to dominate at 45 min.

### 3.2. Strength and Microstructure of Backfill

**Figure 10:**
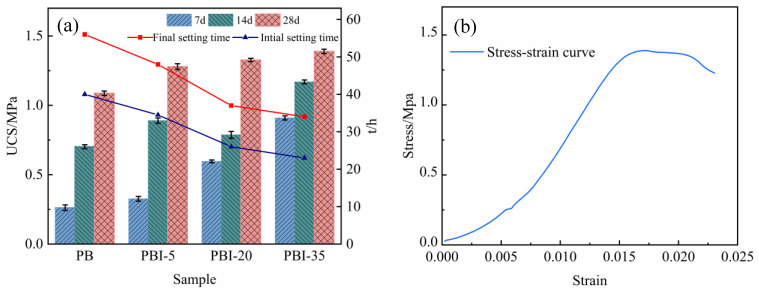

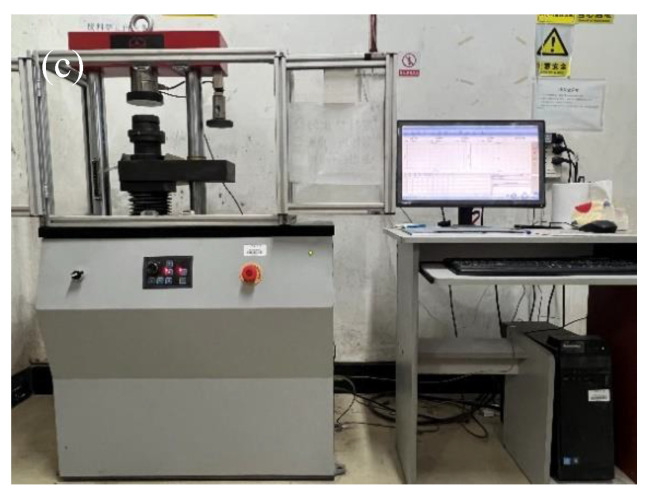
(**a**) UCS and setting time changes of backfill with different amounts of ITs; (**b**) Stress–strain curve of PBI-35 (28 d); (**c**) Servo pressure testing machine for UCS.

The development of the early strength has an important relationship with the setting time of the backfill. The initial and final setting times (IST and FST) of the backfill with various proportions of added ITs are shown in Figure 10. The more ITs added, the shorter the IST and FST of the backfill. When the proportion of ITs added was 5%, the IST and FST of the backfill were 34.5 h and 48 h, respectively, while the IST and FST of the control group were 40 h and 56 h, respectively, representing reductions of 13.8% and 16.7%, respectively. When the amount of ITs added increased to 20%, the IFT and FST of the backfill were 26 h and 37 h, respectively, representing reductions of 35% and 33.9% compared with the control group, respectively. This is because phosphate interferes with the hydration process, prolonging the IFT and FST. The introduction of ITs effectively enhanced the phosphate removal efficiency in a short time, thereby improving the hydration process and shortening the setting time of the cemented PG backfill. 

**Figure 11:**
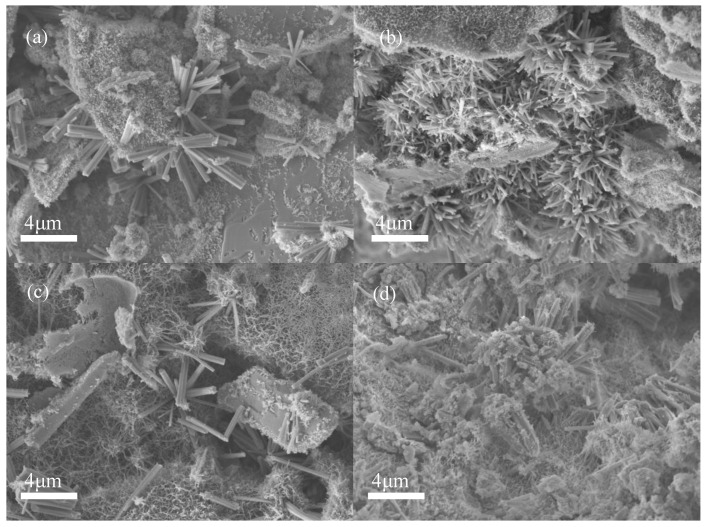
SEM images of backfill samples: (**a**) PB cured for 7 d with ITs; (**b**) PBI-20 cured for 7 d; (**c**) PB cured for 28 d; (**d**) PBI-20 cured for 28 d.

The strength of the backfill plays a decisive role in maintaining the stability of mines [61,62]. The UCS test was conducted on the backfill with various proportions of ITs added, as shown in Figure 10. Generally speaking, the strength of all of the backfills developed well, with the lowest UCS at 28 d being above 1 MPa. The addition of ITs effectively promoted the strength development, especially in the early stages. When cured for 7 days, the UCS of the backfill without ITs was 0.26 MPa. The UCS of the backfill with 5% ITs was slightly improved to 0.32 MPa. When the proportions added were increased to 20% and 35%, the UCS of the backfill reached 0.60 MPa and 0.91 MPa, respectively, which are 2.3 and 3.5 times higher than that of the control group, respectively. The SEM of the backfill cured for 7 days is shown in Figure 11. Without the addition of ITs, a relatively small amount of hydration products was sparsely dispersed on the surface of the PG crystal (Figure 11a), corresponding to the low UCS observed in Figure 10. In comparison, with the addition of 20% ITs, the hydration products in the backfill were densely distributed and mutually supported (Figure 11b), helping to improve the strength of the backfill. 

The 28 d UCS of cemented PG backfill was also enhanced by ITs. As shown in Figure 10, without ITs, the UCS of the control group was 1.08 MPa at 28 d of curing. However, the UCS of the experimental groups with the addition of 5%, 20%, and 35% ITs was 1.20, 1.33, and 1.38 MPa (representing increases of 11.1%, 23.1%, and 27.8%), respectively. Figure 11 shows SEM images of the backfill cured for 28 days. The hydration products in the backfill without ITs were densely distributed on the surface of the PG crystal (Figure 11c); however, the backfill with 20% ITs had more hydration products, which filled the gaps between aggregates and, thus, formed a whole body (Figure 11d), resulting in higher UCS than the control group. 

**Figure 12:**
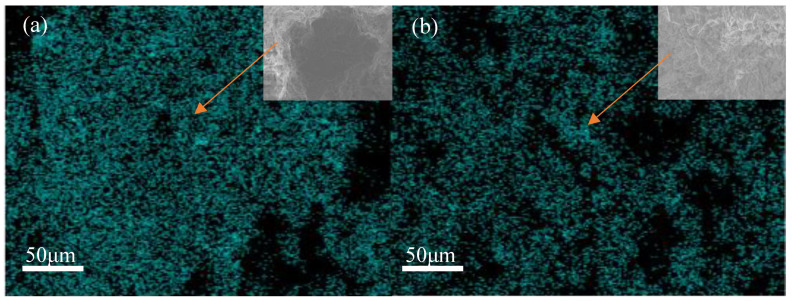
(**a**) The phosphorus mapping diagram of PB cured for 28 d; (**b**) the phosphorus mapping diagram of PBI-20 cured for 28 d.

In order to determine the distribution of phosphate in the backfill, EDS mapping was performed on the backfill without ITs and with 20% ITs, as shown in Figure 12. The results clearly showed that the content of elemental phosphorus was much less in the backfill with 20% ITs. This is because the phosphorus was firmly adsorbed by the porous structure of the ITs, thereby reducing the phosphorus content on the surface of the backfill. Zhou et al. [63] found that high concentrations of phosphate significantly degraded the UCS of cemented PG backfill, due to the consumption of Ca^2+^ and OH^−^ derived from the hydration of the binder. When the ITs were introduced into the cemented PG backfill system, the phosphate in the PG aggregate was quickly and firmly adsorbed onto the ITs by ligand exchange, precipitation, and electrostatic adherence. Without the interference of phosphate, more Ca^2+^ and OH^−^ could participate in the hydration on reaction and produce more hydration products to fill the gaps between aggregates, which benefited the strength development of the backfill. Macroscopically, the slurry was easier to coagulate, and the UCS of the backfill was effectively improved, as discussed above.

### 3.3. Toxic Leaching Test of PG Cemented Backfill 

In order to explore the stability of phosphate immobilized by ITs, the backfill cured for 28 d was selected for toxicity leaching tests. With various proportions of ITs in the cemented PG backfill, the phosphate concentration in the leachates was always lower than 0.02 mg/L—far below the discharge standard (0.5 mg/L). This shows that the immobilization of phosphate in the backfill is not disturbed after adding ITs. Therefore, adding ITs to the PG aggregate can solidify the phosphate in the backfill for a long time, which is of great significance for environmental protection.

After the PG aggregate was treated with the ITs as proposed in this research, the phosphate could be effectively immobilized within the ITs, rendering the aggregate harmless and useful. The stable existence of phosphate in the backfill also indicates that the impurities could be well immobilized during the preparation of the backfill, reducing the amount of phosphate released into the bleeding water and the leachates. This greatly reduces the costs of subsequent wastewater treatment. Furthermore, the immobilization of phosphate can enhance the strength performance of the backfill, with substantial significance for the recycling of solid waste.

## 4. Conclusions

In this study, ITs were used as additives to adsorb phosphate in the process of producing cemented PG backfill. The phosphate immobilization mechanism and the effect of the addition of ITs on the strength of the backfill were investigated. The following conclusions can be drawn:

(1) The addition of ITs to the cemented PG backfill slurry effectively accelerated the removal of phosphate. When the proportion of ITs was 20% or more, the phosphate concentration in the bleeding water could drop to below the discharge standard (0.5 mg/L) within 15 min. The phosphate concentration in the toxic leaching solution of all test blocks was lower than 0.02 mg/L—far lower than the national standard (0.5 mg/L)—indicating that the addition of ITs would not affect the long-term environmental behavior of the backfill.
(2) The linear correlation coefficient in the pseudo-second-order kinetic model was 0.99, indicating that the adsorption of phosphate by ITs takes place through a chemical adsorption process, and there is a strong bond between molecules. ITs are more consistent with Langmuir isotherm model, indicating that the predominant adsorption mode is monolayer adsorption. During the preparation of the backfill, the actual decrease in phosphate attributed to ITs was close to the theoretical values.
(3) The addition of ITs positively affected the UCS of the backfill. With the addition of 20% ITs, the IFT and FST of the backfill were reduced by 35% and 33.9%, respectively, and the 28 d UCS was improved by 23.1%, compared to the control group. Furthermore, it is recommended to study the transportable properties of IT-based cemented PG backfill in the future.
(4) Due to their low cost and good phosphate adsorption performance, ITs have the potential to be used in the preparation of cemented PG backfill to control phosphate pollution, providing new insights into the economical and efficient recycling of solid waste.

## Figures and Tables

**Table 1:** The main chemical compositions of ITs, PG and binder.

Chemical Composition	ITs (%)	Binder (%)	PG (%)
Na_2_O	2.73	0.529	0.12
MgO	2.14	1.5	0.23
Al_2_O_3_	12.98	5.8	0.485
SiO_2_	52.83	18.69	4.393
P_2_O_5_	1.15	0.184	3.952
SO_3_	0.794	4.193	45.68
K_2_O	3.763	1.1	0.0966
CaO	4.682	58.69	35.22
TiO_2_	1.844	0.562	0.0548
Fe_2_O_3_	11.47	3.263	0.245
CuO	0.3888	-	0.006
F	-	-	0.42

**Table 2:** Mix proportions of the cement PG backfill slurry.

Batch No.	PG (%)	ITs (%)	Binder (%)	Water (%)	ITs Proportion to PG (%)
PBS	14.2	0	2.8	83	0
PBSI-5	14.2	0.71	2.8	83	5
PBSI-20	14.2	2.84	2.8	83	20
PBSI-35	14.2	4.98	2.8	83	35

**Table 3:** Compositions of the cement PG backfills.

Batch No.	Aggregate		Binders (%)	Water (%)	ITs proportion to PG (%)
	PG (%)	ITs (%)			
PB	48	0	12	40	0
PBI-5	45.6	2.4	12	40	5
PBI-20	38.4	9.6	12	40	20
PBI-35	31.2	16.8	12	40	35

**Table 4:** Parameters of the Langmuir and Freundlich isotherms for the adsorption of phosphate onto ITs.

Langmuir			Freundlich		
*Q_m_*/(mg/kg)	*K_L_*/(L/mg)	*R* ^2^	*K_F_/*(mg/kg)	1*/n*	*R* ^2^
163.93	0.43	0.985	34.06	0.49	0.898

## Data Availability

Not applicable.
